# Optimal Time Assignment Policy for Maximizing Throughput in Cognitive Sensor Network with Energy Harvesting

**DOI:** 10.3390/s18082540

**Published:** 2018-08-03

**Authors:** Hao Wu, Yong Chen

**Affiliations:** The 63rd Institute, National University of Defense Technology, Nanjing 210007, China

**Keywords:** energy harvesting, cognitive sensor network, time assignment, spectrum sensing

## Abstract

A cognitive sensor network with energy harvesting (EH-CSN) is a promising paradigm to address the issues both in spectrum efficiency and in energy efficiency. The cognitive sensors (CSs) equipped with energy harvesting devices are assumed to operate in a harvesting-sensing-transmission mode and permitted to access the idle licensed frequency bands without causing any harmful jamming to the primary user. By identifying the time fractions of harvesting, sensing, and transmission, we can discuss some design considerations for the EH-CSN. In the meantime, considering the possibility that the primary user may reoccupy the idle channel during the CS’s data transmission duration, we formulate an optimization problem to maximize the average throughput of EH-CSN under a collision constraint and an energy constraint. After deriving the lower and upper bounds of the time fraction for energy harvesting, the uniqueness and existence of the optimal time fraction set have been proved. Finally, our theoretical analysis is also verified through numerical simulations.

## 1. Introduction

Spectrum-efficiency and energy-efficiency have attracted considerable attention for the future wireless communication networks. A cognitive sensor network with energy harvesting (EH-CSN) is a promising paradigm that can be used for these two purposes because of its capability to dynamically access the idle licensed frequency bands and harvest energy from ambient sources. With the successful applications of wireless sensor networks in spectrum monitoring, disaster warning, fire preventing and environmental monitoring, some problems have also exposed, such as the limitations for energy supply and spectrum resources. The sensor nodes are usually facing the extreme complex terrain environment in the above application scenes. Thus, it is hard to supply consistent current for the sensor nodes. The energy harvesting technology will become an efficient method to prolong the survivability of the sensor nodes. In addition, crowded spectrum occupying makes it hard to allocate the new spectrum resources for wireless sensor networks. Then, through combining the cognitive radio technology, wireless sensor network are permitted to access the licensed spectrum band and raise the utilization rate of the spectrum resource.

To improve the spectrum-efficiency, spectrum sensing is adopted to guarantee that the cognitive sensors (CSs) access the under-utilized channels without causing any harmful interference to the primary user (PU). To prolong the lifetime of cognitive sensor network (CSN) and overcome the energy limitation of the CSs, energy harvesting technology is used to provide sufficient energy to fuel the CSN. Moreover, energy-efficient designs [1,2,3,4,5,6,7] and energy harvesting [8,9,10,11,12] represent the two important directions for solving the energy limitation for CSs. Energy-efficient designs can be classified into four groups [13]: minimizing energy consumption, optimizing sensing parameters (e.g., the number of cooperating nodes, detection thresholds, sensing and transmission duration, transmission power), selection and application of fusion and decision rules, and energy-efficient network organization. However energy-efficient designs cannot solve the energy limitation of the CSN essentially.

Energy harvesting is an emerging technology, which can convert the ambient sources (e.g., thermal, vibration, solar, acoustic, wind, or radio frequency energy) into the electric energy to provide the sustainable power supplies for CSs, and has attracted considerable attention of many researchers. Thus, EH-CSN can provide an efficient way to address the issues both in spectrum efficiency and in energy efficiency for the future wireless networks. In general, the designers aim to optimize one or more of sensing parameters in order to maximize the metrics of system performance (e.g., energy efficiency, throughput). In order to guarantee a sufficient protection for PUs, CSs have to periodically sense the status of PUs within a time frame. In the meantime, taking the energy harvesting into consideration, the time frame can be divided into three parts: harvesting duration, sensing duration and transmission duration. Therefore, how to design the time assignment policy for EH-CSN while keeping the high throughput is an emerging problem, which needs to be studied in future wireless communications.

### 1.1. Related Work

Some recent studies [14,15,16,17,18,19,20,21,22,23] have focused on exploiting and investigating the performance of wireless communication systems with energy harvesting. Time resources, sensing parameters and power resources have become very important issues on optimizing the system performance.

In [14], the authors formulated an optimization problem to maximize the harvested energy based on the collision constraint. They finally derived the optimal sensing time. The authors established the model that the cognitive node sensed the presence of PU and harvested radio frequency energy during the sensing time. The authors in [15] considered maximizing the achievable throughput and formulated an optimization problem as a mixed-integer nonlinear programming one. The work [15] put forward the model based on the hypothesis that the false-alarm or miss-detection would be generated by CSs when executing spectrum sensing. Based on detection threshold, the authors in [16] divided the energy harvesting system into two states: a spectrum-limited regime and an energy-limited regime. The optimal detection threshold and spectrum sensing policy are derived to maximize throughput. In [17], the authors proposed a channel selection criterion to maximize the average spectral efficiency for an energy-harvesting cognitive radio network (CRN) under energy constraint. The authors in [18] also considered an energy-harvesting CRN and designed the optimal sensing duration and the sensing threshold together to maximize the average throughput of the secondary network under energy causality constraint and collision constraint. The authors in [19] proposed a wireless EH protocol for a decode-and-forward cognitive relay network with multiple PU transceivers. In this protocol, the secondary nodes could harvest energy from the primary network while sharing the licensed spectrum of the PU. The authors in [20] considered a cluster-based collaborative spectrum sensing scheme in the energy harvesting cognitive wireless communication network. They aimed to maximize the average throughput by identifying the optimal parameter set, including the durations of energy harvesting and spectrum sensing, local detection threshold, and the number of CSs. The authors in [21] considered a centralized collaborative spectrum sensing for an energy-harvesting CRN and formulated the optimization problem to maximize the expected throughput. Based on the finite-horizon partially observable Markov decision process (POMDP), they derived the dynamic sensing access policy. The authors in [22] aimed to investigate the impact of sensing probability, access probability, and energy queue capacity on the maximum achievable throughput. They proposed a two-step opportunistic spectrum access for CRN with energy harvesting and derived the sensing probability. The authors in [23] also formulated the optimization problem to maximize the area throughput of CRN under the performance constraint of primary network. They adopted the stochastic geometry theory to analyze system performance and proposed an efficient algorithm to allocate the bandwidth and time resources for facilitating both the EH and data transmission.

However, the previous works [14,15,16,17,18,19,20,21,22,23] did not consider the case that the PU may reoccupy the idle channel during the CS’s data transmission time. According to this master of literature, only a small amount have taken the case mentioned above into account in designing the energy-efficient spectrum sensing scheme. The authors in [4] considered a framework to jointly optimize design parameters (sensing duration, transmission duration, and the number of cognitive users) that maximized the energy-efficiency. On the basis of the [4], the authors in [5] optimized transmission power and sensing time to maximize energy efficiency and proposed an iterative algorithm to reduce the complexity of solving the optimization problem. The previous works [4,5] both considered possibility of resuming PU activity during transmission period of the CSs. However, they did not use the energy harvesting to provide the energy supplies for CSs.

### 1.2. Contribution

The contribution of this paper is twofold. First, we introduce the Harvesting-Sensing-Transmission (HST) framework for EH-CSN. Within this framework, to guarantee high quality of service (QOS) for CSs, we consider the case that the PU may reoccupy the idle channel during the CS’s data transmission time. We jointly optimize harvesting time, sensing time and transmission time to maximize the throughput of EH-CSN under the energy causality and collision constraint. In order to reduce the analytic complexity of the proposed problem and to improve the convergence speed of the computation, the optimized problem is formulated as a function based on two time fractions, which will represent the harvesting time, sensing time and transmission time.

In addition, by exploiting the hidden constraints of the optimization problem, we derive the exact lower and upper bounds of the time fraction β. Based on the derived interval of β, we prove the uniqueness and existence of the optimal $\left(\alpha^{*},\beta^{*}\right)$. Through mathematical reformulation, we derive the theoretical expression to determine the optimal $\alpha^{*}$ and $\beta^{*}$. An iterative algorithm is proposed to determine the optimal design parameters for CSN.

This remainder of this paper is organized as follows: Section 2 introduces our system model. Problem formulation and solution is presented in Section 3. Simulation results are presented and discussed in Section 4. Finally, our conclusions are provided in Section 5.

## 2. System Model

Considering a EH-CSN comprised of a pair of energy harvesting enabled CS transceivers and a pair of power-supplied PU transceivers. The EH-CSN is assumed to operate in a time-slotted fashion. The CS stores the harvested energy in its energy storage device (e.g., a super capacitor). Such a device cannot charge and discharge simultaneously, and the energy storage device stops working while CS turns on spectrum sensing or data transmission. That is, the CS is assumed to operate in a “energy half-duplex mode” [24]. Moreover, compared to the harvested energy, the battery capacity is assumed to be infinite, which can avoid the energy overflow. The CS is permitted to access the licensed frequency band not occupied by the PU opportunistically. Consider $\pi_{0}$ to be the probability that a PU is active and $\pi_{1}$ be the probability that a PU is passive. $\pi_{0}+\pi_{1}=1$, $\pi_{0}$ and $\pi_{1}$ can be found based on the long-term measurements. According to [4,25], the durations of PUs’ states are assumed to be an exponential distribution with averages of $\tau_{0}$ and $\tau_{1}$. Then, π0=τ0τ0τ0+τ1τ0+τ1 and π1=τ1τ1τ0+τ1τ0+τ1.

The time-slotted duration *T* is assumed to be divided into three parts: harvesting duration αT, sensing duration βT and transmission duration $\left(1-\alpha -\beta \right)T$, where α is the time fraction of harvesting and is defined as the ratio of harvesting duration to *T*, β is the time fraction of sensing and is defined as the ratio of sensing duration to *T*. Then, the probability that PU may resume its activity when CS transmits data can be expressed as $P_{I}=1-e^{-\frac{\left(1-\alpha -\beta \right)T}{\tau_{0}}}$. The average rate of energy harvesting and the average energy consumed rate of sensing are assumed to be $e_{H}$ (J/s) and $e_{S}$ (J/s), respectively. Moreover, the $e_{H}$ and $e_{S}$ will take some unchanged values at a specific time or in a specific area. Moreover, the battery has a storing efficiency $\eta \left(0⩽\eta ⩽1\right)$. The energy consumption of the CS should not exceed the harvested energy within a time slot, that is $\eta e_{H}\alpha T⩾e_{S}\beta T$, which indicates the energy constraint.

If the sampling frequency is $f_{s}$ and an energy detector is adopted by CS to calculate the energy statistic over the sensing duration βT, the number of sampling points is $L=\beta T\times f_{s}$. A PU’s transmitted signal is assumed to be an independent and identically distributed (iid) random process with zero mean and variance $\sigma_{x}^{2}$. The noise is also assumed to be a real-valued Gaussian variable with zero mean and variance $\sigma_{n}^{2}$. The received signal power at the CS’s transmitter is $\sigma_{r}^{2}=\left|h_{pc}\right|\sigma_{x}^{2}$, and $h_{pc}$ is the channel gain from PU’s transmitter to CS’s transmitter. If the *k*-th sampling point of the CS is $y\left(k\right)$, then the energy statistic established by CS is $S=\frac{1}{L}\sum_{k=1}^{L}{\left|y\left(k\right)\right|}^{2}$. When the sampling points are sufficient, *S* follows the Gauss distribution because of the central limit theorem (CLT). fxxHθHθ, θ∈0,1 is assumed to be a probability density function of *S* under the hypotheses $H_{0}$ and $H_{1}$, and

(1) fx/H0=e−x−σn24σn4/L2π2σn4/L, 0≤x<∞,fxxH1H1=e−x−σn2−σr24σr2+σn224σr2+σn22LL2π2σr2+σn222σr2+σn22LL, 0≤x<∞.

Nevertheless, formula (1) is obtained by satisfying the condition that the received signal and noise are both real valued. If they are both circularly symmetric and complex valued, the parameters of the probability density function will be changed. However they are outside the scope of our discussion.

Then, the detection probability $P_{d}$ and the false alarm probability $P_{f}$ are given by

(2) Pd=Qλ−σn2+σr2σn2+σr2σn2+σr2βTfsβTfs22βTfsβTfs22,Pf=Qλ−σn2σn2σn2βTfsβTfs22βTfsβTfs22,

where λ is the detection threshold. From formula (2), we know that the $P_{d}$ and $P_{f}$ interact with each other. It is hard to obtain the highest detection probability while keeping the lowest false alarm probability. We usually need to design the appropriate sensing parameters for achieving the trade-off between the detection probability and false alarm probability.

In our proposed model, the battery capacity is assumed to be infinite and to be the ideal one without any energy losses. If the battery capacity is finite, we will add another constraint which avoids the energy overflow, thus making the performance analysis intractable. In addition, the proposed model assumes that the harvested energy will be consumed completely in each time slot. However, in some scenes, in order to ensure the reliability of the data transmitting, the transmitted power needs to reach a certain value. In that case, we will add another constraint that satisfies the requirement for the transmitted power. Moreover, if the sensor node is assumed to operate in a “energy full-duplex mode”, we need to redesign the assignment policy and reconsider the optimization object. Whether the above-mentioned more complicated models would affect the assignment policy of time resource and energy resource needs to be studied more in depth in a new study, and is beyond the scope of this paper.

## 3. Problem Formulation and Solution

An important metric for the system performance of a CSN is average throughput. According to the [26], we know that CS’s achievable throughput consists of two parts: $C_{0}$ and $C_{1}$, where $C_{0}$ is the volume of data transmitted during the transmission duration when the PU is absent and $C_{1}$ is the volume of data transmitted during the transmission duration when the PU is present. $C_{1}$ is not considered since CS would be collided with PU, which results in the high data error rate at CS’s receiver. The $C_{1}$ is very small compared to the $C_{0}$. In this paper, we will only take $C_{0}$ into account rationally. If no false alarm is generated at CS’s transmitter over sensing duration βT, $C_{0}$ can be given by

(3) $$\begin{array}{c} C_{0}=\frac{\left(1-\alpha -\beta \right)T}{T}\log_{2}\left(1+\gamma_{cs}\right), \end{array}$$

where $\gamma_{cs}$ denotes the ratio of the received power to the noise power at the CS’s receiver. The energy left for data transmission can be expressed as $\eta e_{H}\alpha T-e_{S}\beta T$. Then, the $\gamma_{cs}$ can be expressed as

(4) $$\begin{array}{c} \gamma_{cs}=\frac{\left|h_{cs}\right|\left(\eta e_{H}\alpha T-e_{S}\beta T\right)}{\sigma_{n}^{2}\left(1-\alpha -\beta \right)T}, \end{array}$$

where $h_{cs}$ is the channel gain from CS’s transmitter to CS’s receiver. It must be noted that the propagation issues will affect the optimal energy harvesting time, sensing time and transmission time. However, no matter what value the propagation issue is set to, the method to analyze the optimization objective keeps the same. For simple analyticity, the propagation issue $h_{cs}$ gets the value 1. If the probability of false alarm and the possibility that the PU may reoccupy the idle channel during the CS’s data transmission are taken into account for investigating the achievable throughput. The average achievable throughput *R* of the CS in each time-slot is given by

(5) $$\begin{array}{c} R=C_{0}\left(1-P_{f}\right)\pi_{0}\left(1-P_{I}\right). \end{array}$$

From formula (5), one can see that α and β are the only two parameters which affect the system performance of EH-CSN in our proposed scheme. The harvesting duration, sensing duration, and transmission duration are completely controlled by α and β. Finally, the goal of this paper is to design α and β together with an eye toward maximizing the achievable throughput under the collision constraint and energy constraint. Then, we formulate the optimization problem as follows:

(6) $$\begin{array}{c} \max_{\left\{\alpha ,\beta \right\}}C_{0}\left(1-P_{f}\right)\pi_{0}\left(1-P_{I}\right),\\ s.t.\;\;\;P_{d}\geq {\bar{P}}_{th},\\ \;\;\;\eta e_{H}\alpha T>e_{S}\beta T,\\ \;\;\;\alpha +\beta <1,\\ \;\;\;\alpha >0,\;\beta >0, \end{array}$$

where ${\bar{P}}_{th}$ is the target detection probability, which represents the collision constraint. Choosing the $P_{d}={\bar{P}}_{th}$, the detection threshold can be expressed as λ=σr2+σn21+Q−1P¯thβTfsβTfs22. From the section “Optimality conditions” in [27], the Karush–Kuhn–Tucker (KKT) optimality conditions and complementary slackness are adopted in this optimization problem. It is not necessary for us to discuss the convexity of formula (6) with respect to α and β. Using Lagrange multipliers, we can transform the constrained optimization problem into an unconstrained optimization problem as follows:

(7) $$\begin{array}{c} \psi \left(\alpha ,\beta \right)=-C_{0}\left(1-P_{f}\right)\pi_{0}\left(1-P_{I}\right)+\mu_{1}\left(e_{S}\beta T-\eta e_{H}\alpha T\right)+\mu_{2}\left(\alpha +\beta -1\right)-\mu_{3}\alpha -\mu_{4}\beta \end{array},$$

where $\mu_{1},\;\mu_{2},\;\mu_{3},\;\mu_{4}$ are non-negative Lagrange multipliers.

Differentiating $\psi \left(\alpha ,\beta \right)$ with respect to α and β respectively, we can derive that

(8) $$\begin{array}{c} \frac{d\psi \left(\alpha ,\beta \right)}{d\alpha }=C\left(\alpha ,\beta \right)F\left(\beta \right)\theta \left(\alpha ,\beta \right)\pi_{0}-\frac{F\left(\beta \right)\theta \left(\alpha ,\beta \right)}{\ln2\left[\sigma_{n}^{2}\left(1-\alpha -\beta \right)+\eta e_{H}\alpha -e_{S}\beta \right]}\left(\eta e_{H}-\eta e_{H}\beta -e_{S}\beta \right)\pi_{0}\\ \;+\left(\alpha +\beta -1\right)C\left(\alpha ,\beta \right)F\left(\beta \right)\theta \left(\alpha ,\beta \right)\frac{\pi_{0}T}{\tau_{0}}-\mu_{1}\eta e_{H}T+\mu_{2}-\mu_{3} \end{array}$$

(9) $$\begin{array}{c} \frac{d\psi \left(\alpha ,\beta \right)}{d\beta }=C\left(\alpha ,\beta \right)F\left(\beta \right)\pi_{0}\theta \left(\alpha ,\beta \right)+\frac{\left(e_{S}-\eta e_{H}\alpha -e_{S}\alpha \right)F\left(\beta \right)\pi_{0}\theta \left(\alpha ,\beta \right)}{\ln2\left[\sigma_{n}^{2}\left(1-\alpha -\beta \right)+\eta e_{H}\alpha -e_{S}\beta \right]}\\ +\exp\left(\frac{-{\left(A\sqrt{\beta }+B\right)}^{2}}{2}\right)\times \frac{\left(\alpha +\beta -1\right)C\left(\alpha ,\beta \right)A\pi_{0}}{\sqrt{2\pi }\times 2\sqrt{\beta }}\theta \left(\alpha ,\beta \right)\\ +\left(\alpha +\beta -1\right)C\left(\alpha ,\beta \right)F\left(\beta \right)\pi_{0}\theta \left(\alpha ,\beta \right)\frac{T}{\tau_{0}}+\mu_{1}e_{S}T+\mu_{2}-\mu_{4}, \end{array}$$

where

(10) γ=σr2σn2, A=γTfsTfs22, B=Q−1P¯th1+γ,Cα,β=log21+ηeHα−eSβσn21−α−β,Fβ=1−Pf=1−QAβ+B,θα,β=1−PI=e−1−α−βTτ0,

and the complementary slackness conditions are given by

(11) $$\begin{array}{c} \mu_{1}^{*}\left(e_{S}\beta^{*}T-\eta e_{H}\alpha^{*}T\right)=0,\\ \mu_{2}^{*}\left(\alpha^{*}+\beta^{*}-1\right)=0,\\ \mu_{3}^{*}\alpha^{*}=0,\;\mu_{4}^{*}\beta^{*}=0,\\ \mu_{1}^{*}\geq 0,\;\mu_{2}^{*}\geq 0,\;\mu_{3}^{*}\geq 0,\;\mu_{4}^{*}\geq 0. \end{array}$$

In EH-CSN, $e_{S}\beta^{*}T-\eta e_{H}\alpha^{*}T=0$ denotes that the harvested energy is completely consumed by spectrum sensing, it will not leave any energy for data transmission. $\alpha^{*}+\beta^{*}-1=0$ shows that the duration of data transmission is zero. $\alpha^{*}=0$ shows that the duration of energy harvesting is zero, and $\beta^{*}=0$ shows that the duration of spectrum sensing is zero. If any of the above-mentioned four situations occur, EH-CSN will not work properly. Thus, in our optimization problem, the optimal Lagrange multipliers $\mu_{1}^{*},\;\mu_{2}^{*},\;\mu_{3}^{*},\;\mu_{4}^{*}$ always need to get zero.

**Theorem** **1.** *There exists an unique optimal set $\left(\alpha^{*},\beta^{*}\right)$ that maximizes the achievable throughput, where $\beta^{*}\in \left[\beta_{1},\beta_{2}\right]$ and $\beta_{1},\;\beta_{2}$ meet the conditions $\rho \left(\beta_{1}\right)=1$ and $\xi \left(\beta_{2}\right)=0$, respectively. In the meantime, $\beta^{*}$ meets the following equation:*

(12) $$\begin{array}{c} \ln\left(1-\frac{\eta e_{H}}{\sigma_{n}^{2}}+\frac{\eta e_{H}-\eta e_{H}\beta -e_{S}\beta }{\sigma_{n}^{2}\frac{\tau_{0}}{T}\left(1-\rho \left(\beta \right)\right)}\right)=\frac{1}{\rho \left(\beta \right)}\times \frac{\eta e_{H}-\eta e_{H}\beta -e_{S}\beta }{\left(\sigma_{n}^{2}-\eta e_{H}\right)\frac{\tau_{0}}{T}\left(1-\rho \left(\beta \right)\right)+\eta e_{H}-\eta e_{H}\beta -e_{S}\beta }. \end{array}$$

*The optimal $\alpha^{*}=1-\beta^{*}-\frac{\tau_{0}}{T}\left(1-\rho \left(\beta^{*}\right)\right)$, where*

(13) $$\begin{array}{c} \rho \left(\beta \right)=\frac{\eta e_{H}-\eta e_{H}\beta -e_{S}\beta }{F\left(\beta \right)\left(\eta e_{H}+e_{S}\right)}\times \frac{1}{\sqrt{2\pi }}\times \exp\left(\frac{-\left(A\sqrt{\beta }+B\right){}^{2}}{2}\right)\times \frac{A}{2\sqrt{\beta }}\\ \xi \left(\beta \right)=\rho \left(\beta \right)-\frac{T}{\tau_{0}}\left(1+\frac{e_{S}}{\eta e_{H}}\right)\beta +\frac{T}{\tau_{0}}-1. \end{array}$$

**Proof.**  See Appendix A. ☐

Then, we propose Algorithm 1 to determine the optimal $\left(\alpha^{*},\beta^{*}\right)$ that maximizes the achievable throughput. Bisection is firstly used to numerically find the lower bound $\beta_{1}$, the upper bound $\beta_{2}$ and the optimal $\beta^{*}$, and it is denoted as $bis\left(f\left(x\right),\;g\left(x\right),\;x_{1},\;x_{2}\right)$, where $f\left(x\right)$ and $g\left(x\right)$ are the functions that we want to determine the root of $f\left(x\right)=g\left(x\right)$, while $x_{1},\;x_{2}$ are the lower and upper bounds to which the root belongs.

**Figure 1:**
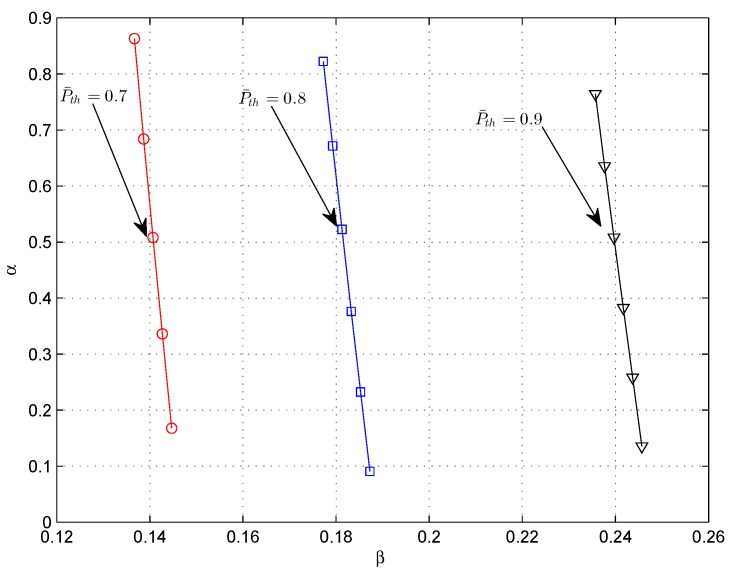
β vs. the corresponding α, for different ${\bar{P}}_{th}$.

The algorithm firstly reduces the interval which the sensing fraction β belongs to. This step provides an advantage of speeding up the searching rate. In addition, the derived lower and upper bounds for β guarantee the unique root of the equation $\varsigma \left(\beta \right)\;=\;\upsilon \left(\beta \right)$. The proposed algorithm is designed based on the existence and uniqueness of the optimal sensing fraction β, which has been proved in Appendix A. The relationship between α and β is expressed as $\alpha =1-\beta -\frac{\tau_{0}}{T}\left(1-\rho \left(\beta \right)\right)$, which is demonstrated in Figure 1.

Based on the assumption that the durations of PU’s states are assumed to be an exponential distribution, we derive the optimal time assignment policy and propose the efficient algorithm to determine the optimal design parameters for CSN. However, we need to know that the durations of PUs’ states may not always follow the exponential distribution and do not have the memoryless property in many actual application scenes. When PU busy periods are correlated, for example, it follows log-normal distribution, then the probability that PU may resume its activity when CS transmits data can be expressed as another complicated formula. As result, it is necessary for us to adopt another method to solve the optimization problem in that case. In the meantime, we may hardly determine the exact lower and upper bounds of the time fraction. Moreover, it may be hard to prove the uniqueness and existence of the optimal time set. Compared to our proposed algorithm, the approaches to solve the optimization problem when PU busy periods are correlated may be completely different. Thus, our proposed model is suitable for the PU’s state with exponential distribution. If we encounter other distribution, we need to exploit specific method to solve those problems.
**Algorithm 1** Finding the optimal set $\left(\alpha^{*},\beta^{*}\right)$1:**Input**$f_{s},T,\gamma ,e_{H},e_{S},\tau_{0},{\bar{P}}_{th},\sigma_{n}^{2}$2:**Compute**$\beta_{1}=bis\left(\rho \left(\beta \right),1,0,\frac{\eta e_{H}}{e_{H}+e_{S}}\right)$3:**Compute**$\beta_{2}=bis\left(\xi \left(\beta \right),0,0,\frac{\eta e_{H}}{\eta e_{H}+e_{S}}\right)$4:**Compute**$\beta^{*}=bis\left(\varsigma \left(\beta \right),\upsilon \left(\beta \right),\beta_{1},\beta_{2}\right)$5:**Compute**$\alpha^{*}=1-\beta^{*}-\frac{\tau_{0}}{T}\left(1-\rho \left(\beta^{*}\right)\right)$6:**Return**$\left(\alpha^{*},\beta^{*}\right)$

## 4. Simulations

The above theoretical analysis is verified and shown through numerical simulations in this section. The simulation parameters are summarized in Table 1, where the γ is just the signal-to-noise radio. Taking the solar energy as an example, the harvesting efficiency of solar energy is usually assumed to be 0.5 J/s, which is usually greater than the noise variance. The EH-CSN can obtain the maximum throughput when the time fraction set is $\left(\alpha^{*}=0.1205,\;\beta^{*}=0.2389\right)$ calculated from Algorithm 1. In order to demonstrate the accuracy of the optimal $\alpha^{*}$ and $\beta^{*}$ derived from the Algorithm 1 in Section 3, we consider that α, β both vary in the range $\left[0,1\right]$. In order to avoid the overflow in computation, we must add some constraints to formula (5) for calculating the achievable throughput, that is

(14) $$\begin{array}{c} R=\left\{\begin{array}{c} 0,\;\eta e_{H}\alpha T-e_{S}\beta T\leq 0,\;or\;1-\alpha -\beta \leq 0,\\ C_{0}\left(1-P_{f}\right)\pi_{0}\left(1-P_{I}\right),\;else. \end{array}\right. \end{array}$$

**Figure 2:**
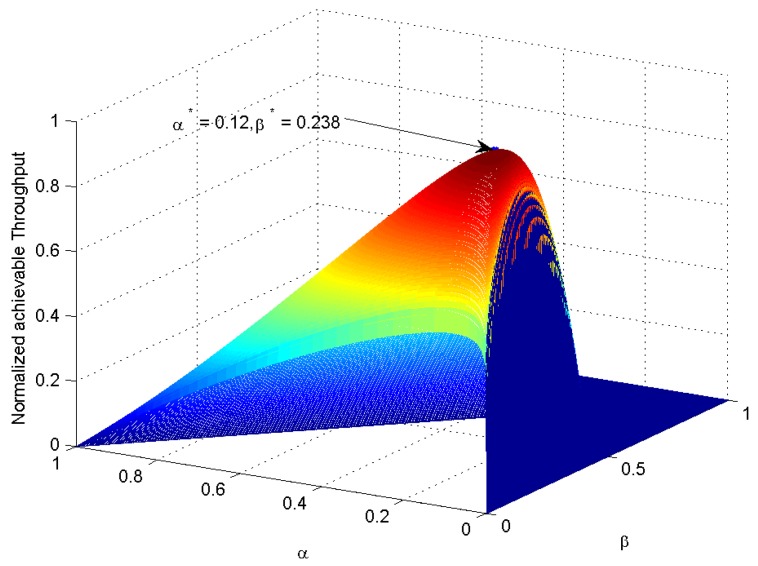
Normalized achievable throughput versus α&β (γ = −16 dB).

Figure 2 plots the normalized achievable throughput against α and β. The exhaustive search method is used to search the maximum point of the normalized throughput. Then, we can get the optimal simulation solution (0.12,0.238) for $\left(\alpha^{*},\beta^{*}\right)$, which perfectly matches with the theoretical solution (0.1205,0.2389) derived from the proposed Algorithm 1. Thus, the proposed algorithm is efficient to determine the optimal time assignment policy.

**Figure 3:**
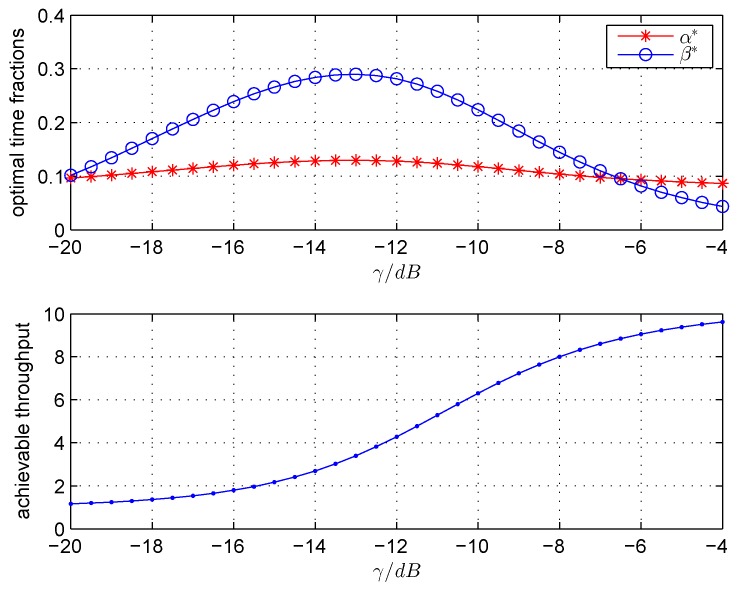
The optimal $\alpha^{*}$, $\beta^{*}$ and achievable throughput versus γ.

**Figure 4:**
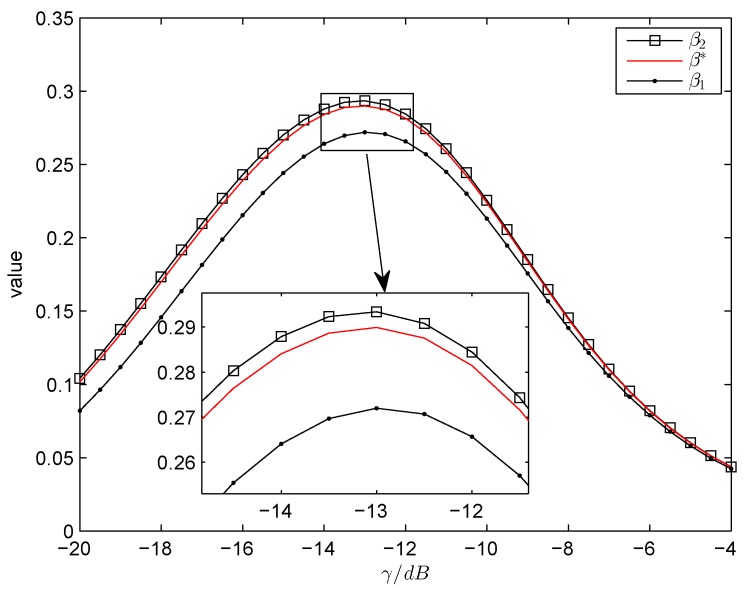
The lower bound $\beta_{1}$, the upper bound $\beta_{2}$ and the optimal $\beta^{*}$ versus γ.

Next, in order to provide a better understanding on how the γ affects the system performance, we take the achievable throughput, the optimal $\alpha^{*}$ and $\beta^{*}$ as a function of γ. Figure 3 plots the optimal $\alpha^{*}$, $\beta^{*}$ and the corresponding achievable throughput against γ respectively. Figure 4 plots the lower bound $\beta_{1}$, the upper bound $\beta_{2}$ and the optimal $\beta^{*}$ versus γ. One can see that the $\beta^{*}$ increases firstly and decreases subsequently as γ increases. In the meantime, the $\alpha^{*}$ changes a little as γ increases. This is because when the γ is lower than a certain value, the CS needs more sensing duration to improve the detection probability, which satisfies the target detection performance. When the γ continues to increase above a certain value, the CS needs less sensing duration to determine the status of PU while keeping the high detection performance. Then, it will leave more time for CS to transmit data, which would lead to the increasing of achievable throughput. However, the γ continues to increase, the residual time $\left(1-\alpha^{*}-\beta^{*}\right)\times T$ will not vary very much, which makes CS obtain the stable throughput.

**Figure 5:**
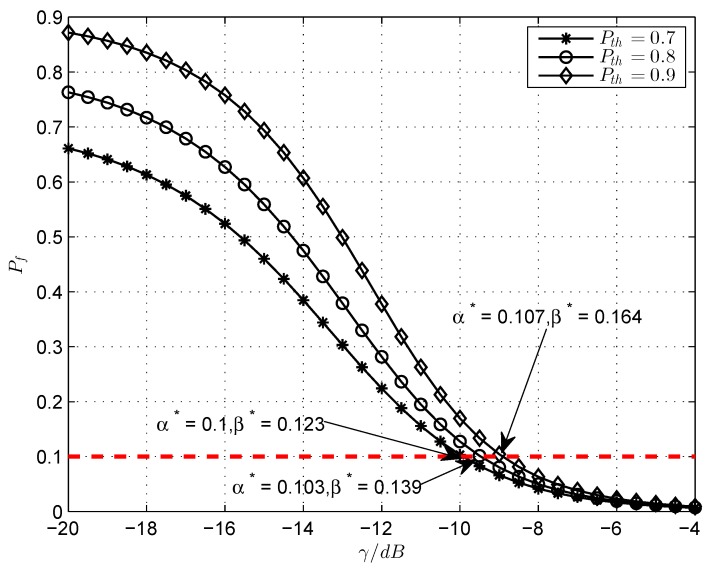
Probability of false alarm $P_{f}$ vs. γ, for different ${\bar{P}}_{th}$.

Finally, the impact of varying γ on the false alarm probability for different ${\bar{P}}_{th}$, at the corresponding optimal $\left(\alpha^{*},\beta^{*}\right)$ is demonstrated in Figure 5. In this paper, the false alarm probability target is not taken as an additional constraint to the optimization problem (6). If the false alarm probability constraint is set to 0.1, which is shown with the red line. It is easily shown that the derived optimal operating point $\left(\alpha^{*},\beta^{*}\right)$ cannot meet the false alarm probability target if the γ is less than a certain threshold value—for example, when ${\bar{P}}_{th}=0.9$, γ=−9 dB just meets the false alarm probability target 0.1 and the corresponding optimal operating point is $\left(\alpha^{*}=0.107,\;\beta^{*}=0.164\right)$. If γ<−9 dB, the false alarm probability target cannot be satisfied at any points.

## 5. Conclusions

In order to design an optimal time assignment policy for EH-CSN, we formulated the optimization problem to maximize the achievable throughput under the collision constraint and the energy constraint. We derived the lower and upper bounds of the time fraction of energy harvesting in the process of solving the optimization problem. Analytical and simulation results showed that there existed a unique time fraction set to maximize the achievable throughput. An efficient algorithm was proposed to find the optimal set of time fractions. Finally, our theoretical analysis is also verified through numerical simulations.

## Figures and Tables

**Table 1:** Simulation parameters

Parameter	Value
Noise variance $\sigma_{n}^{2}$	$10^{-8}$ W
Time-slot duration *T*	50 ms
The average rate of energy harvesting $e_{H}$	0.5 J/s
The average consumed energy rate of sensing $e_{S}$	0.1 J/s
The sampling frequency $f_{s}$	100 kHz
$\tau_{0}$	0.3
$\tau_{1}$	0.2
Storing efficiency η	0.7
γ	−16 dB
The target detection probability ${\bar{P}}_{th}$	0.9

**Figure A1:**
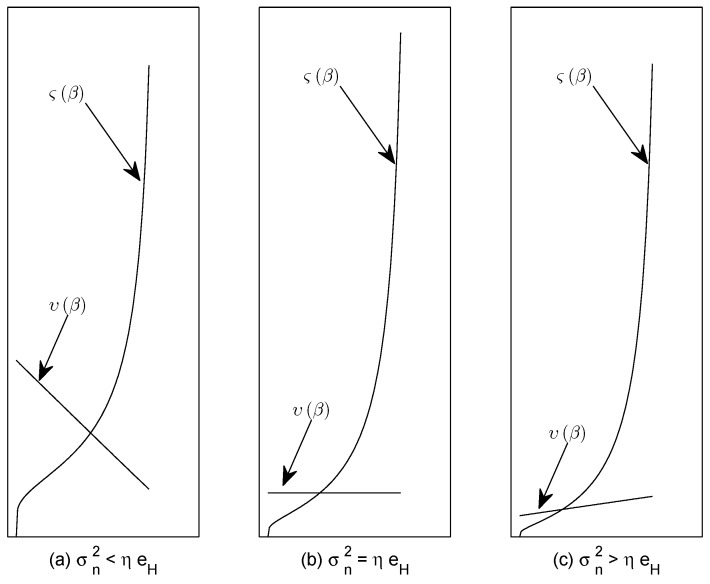
Illustration of $\varsigma \left(\beta \right)$ and $\upsilon \left(\beta \right)$ versus β over the range $\beta_{1}⩽\beta ⩽\beta_{2}$.

## Appendix A. Proof of Theorem 1

Substituting $\mu_{1}^{*}=0,\;\mu_{2}^{*}=0,\;\mu_{3}^{*}=0,\;\mu_{4}^{*}=0$ into $\frac{d\psi \left(\alpha ,\beta \right)}{d\alpha }=0$ and $\frac{d\psi \left(\alpha ,\beta \right)}{d\beta }=0$, we compute $\frac{d\psi \left(\alpha ,\beta \right)}{d\alpha }-\frac{d\psi \left(\alpha ,\beta \right)}{d\beta }=0$ and derive the following expression:

(A1) $$\begin{array}{c} \frac{F\left(\beta \right)\left(\eta e_{H}+e_{S}\right)}{\ln2\left(\sigma_{n}^{2}\left(1-\alpha -\beta \right)+\eta e_{H}\alpha -e_{S}\beta \right)}=\frac{A\times C\left(\alpha ,\beta \right)}{\sqrt{2\pi }\times 2\sqrt{\beta }}e^{\frac{-{\left(A\sqrt{\beta }+B\right)}^{2}}{2}}. \end{array}$$

From $\frac{d\psi \left(\alpha ,\beta \right)}{d\alpha }=0$, we can easily get the following equation:

(A2) $$\begin{array}{c} C\left(\alpha ,\beta \right)=\frac{\frac{1}{\ln2}\times \frac{\eta e_{H}-\eta e_{H}\beta -e_{S}\beta }{\sigma_{n}^{2}\left(1-\alpha -\beta \right)+\eta e_{H}\alpha -e_{S}\beta }}{1+\left(\alpha +\beta -1\right)\frac{T}{\tau_{0}}}. \end{array}$$

Substituting formula (A2) into formula (A1), it is derived that

(A3) $$\begin{array}{c} \frac{\eta e_{H}-\eta e_{H}\beta -e_{S}\beta }{F\left(\beta \right)\left(\eta e_{H}+e_{S}\right)}\times \frac{1}{\sqrt{2\pi }}\times \exp\left(\frac{-{\left(A\sqrt{\beta }+B\right)}^{2}}{2}\right)\times \frac{A}{2\sqrt{\beta }}\;=\;1-\frac{T}{\tau_{0}}\left(1-\alpha -\beta \right). \end{array}$$

Then, the left-hand side of the above equation can be denoted as

(A4) $$\begin{array}{c} \rho \left(\beta \right)=\frac{\eta e_{H}-\eta e_{H}\beta -e_{S}\beta }{F\left(\beta \right)\left(\eta e_{H}+e_{S}\right)}\times \frac{1}{\sqrt{2\pi }}\times \exp\left(\frac{-{\left(A\sqrt{\beta }+B\right)}^{2}}{2}\right)\times \frac{A}{2\sqrt{\beta }}. \end{array}$$

Substituting formula (A4) into formula (A3), we can get the expression of α as follows:

(A5) $$\begin{array}{c} \alpha \;=\;1-\frac{\tau_{0}}{T}\left(1-\rho \left(\beta \right)\right)-\beta \end{array}$$

According to the constraint conditions from the formula (6), that is $\eta e_{H}\alpha T>e_{S}\beta T$ and α+β<1, it is derived that

(A6) $$\begin{array}{c} 0<\beta <\frac{\eta e_{H}}{e_{S}+\eta e_{H}},\\ 1-\beta >\alpha >\frac{e_{S}}{\eta e_{H}}\beta . \end{array}$$

From formula (A3), due to $\frac{T}{\tau_{0}}\left(1-\alpha -\beta \right)>0$, we can derive $\rho \left(\beta \right)<1$. Substituting formula (A5) into $\eta e_{H}\alpha T>e_{S}\beta T$, it is derived that

(A7) $$\begin{array}{c} \rho \left(\beta \right)-\frac{T}{\tau_{0}}\left(1+\frac{e_{S}}{\eta e_{H}}\right)\beta +\frac{T}{\tau_{0}}-1>0. \end{array}$$

Let

(A8) $$\begin{array}{c} \xi \left(\beta \right)=\rho \left(\beta \right)-\frac{T}{\tau_{0}}\left(1+\frac{e_{S}}{\eta e_{H}}\right)\beta +\frac{T}{\tau_{0}}-1. \end{array}$$

Then, $\xi \left(\beta \right)>0$. Obviously, $\rho \left(\beta \right)$ and $\xi \left(\beta \right)$ both decrease with β, which is in the range $0<\beta <\frac{\eta e_{H}}{e_{S}+\eta e_{H}}$. $\beta_{1}$ and $\beta_{2}$ are assumed to satisfy the ${\left.\rho \left(\beta \right)\right|}_{\beta =\beta_{1}}-1=0$ and ${\left.\xi \left(\beta \right)\right|}_{\beta =\beta_{2}}=0$, respectively. From (A8), we can get $\xi \left(\beta \right)-\left(\rho \left(\beta \right)-1\right)=\frac{T}{\tau_{0}}-\frac{T}{\tau_{0}}\left(1+\frac{e_{S}}{\eta e_{H}}\right)\beta$, the right term $\frac{T}{\tau_{0}}-\frac{T}{\tau_{0}}\left(1+\frac{e_{S}}{\eta e_{H}}\right)\beta$ is always greater than zero for $0<\beta <\frac{\eta e_{H}}{e_{S}+\eta e_{H}}$. Thus, we can get $\xi \left(\beta \right)\geq \rho \left(\beta \right)-1$. It can be concluded that $0<\beta_{1}\leq \beta \leq \beta_{2}<\frac{\eta e_{H}}{\eta e_{H}+e_{S}}$.

According to the denotation of $C\left(\alpha ,\beta \right)$ in formula (10) and formula (A2), it is derived that

(A9) $$\begin{array}{c} \log_{2}\left(1+\frac{\eta e_{H}\alpha -e_{S}\beta }{\sigma_{n}^{2}\left(1-\alpha -\beta \right)}\right)=\frac{\frac{1}{\ln2}\times \frac{\eta e_{H}-\eta e_{H}\beta -e_{S}\beta }{\sigma_{n}^{2}\left(1-\alpha -\beta \right)+\eta e_{H}\alpha -e_{S}\beta }}{1-\left(1-\alpha -\beta \right)\frac{T}{\tau_{0}}}. \end{array}$$

Substituting formula (A5) into formula(A9), we can derive that

(A10) $$\begin{array}{c} \ln\left(1-\frac{\eta e_{H}}{\sigma_{n}^{2}}+\frac{\eta e_{H}-\eta e_{H}\beta -e_{S}\beta }{\sigma_{n}^{2}\frac{\tau_{0}}{T}\left(1-\rho \left(\beta \right)\right)}\right)=\frac{1}{\rho \left(\beta \right)}\times \frac{\eta e_{H}-\eta e_{H}\beta -e_{S}\beta }{\left(\sigma_{n}^{2}-\eta e_{H}\right)\frac{\tau_{0}}{T}\left(1-\rho \left(\beta \right)\right)+\eta e_{H}-\eta e_{H}\beta -e_{S}\beta }. \end{array}$$

Through some mathematical processing, Equation (A10) can be expressed as

(A11) $$\begin{array}{c} \frac{1}{\rho \left(\beta \right)\times \ln\left(1-\frac{\eta e_{H}}{\sigma_{n}^{2}}+\frac{\eta e_{H}-\eta e_{H}\beta -e_{S}\beta }{\sigma_{n}^{2}\frac{\tau_{0}}{T}\left(1-\rho \left(\beta \right)\right)}\right)}=1+\left(\sigma_{n}^{2}-\eta e_{H}\right)\frac{\tau_{0}}{T}\frac{1-\rho \left(\beta \right)}{\eta e_{H}-\eta e_{H}\beta -e_{S}\beta }. \end{array}$$

Let

(A12) $$\begin{array}{c} \varsigma \left(\beta \right)=\frac{1}{\rho \left(\beta \right)\ln\left(1-\frac{\eta e_{H}}{\sigma_{n}^{2}}+\frac{\left(\eta e_{H}-\eta e_{H}\beta -e_{S}\beta \right)}{\sigma_{n}^{2}\frac{\tau_{0}}{T}\left(1-\rho \left(\beta \right)\right)}\right)},\\ \upsilon_{1}\left(\beta \right)=\frac{1-\rho \left(\beta \right)}{\eta e_{H}-\eta e_{H}\beta -e_{S}\beta },\\ \upsilon \left(\beta \right)=1+\left(\sigma_{n}^{2}-\eta e_{H}\right)\frac{\tau_{0}}{T}\upsilon_{1}\left(\beta \right). \end{array}$$

Then, we can rewrite Equation (A11) as follows:

(A13) $$\begin{array}{c} \varsigma \left(\beta \right)=\upsilon \left(\beta \right). \end{array}$$

Thus, the optimal $\beta^{*}$ must satisfy the equation $\varsigma \left(\beta \right)=\upsilon \left(\beta \right)$. Then, the proof of the existence and uniqueness for the optimal $\beta^{*}$ will be shown in the subsequent parts.

Because $\rho \left(\beta \right)$ decreases with β, it is easily derived that $\varsigma \left(\beta \right)$ and $\upsilon_{1}\left(\beta \right)$ increase with β for $\beta_{1}⩽\beta ⩽\beta_{2}$. Due to $\lim_{\beta \to \beta_{1}}\varsigma \left(\beta \right)=0,\;\lim_{\beta \to \beta_{2}}\varsigma \left(\beta \right)=+\infty$, $\lim_{\beta \to \beta_{1}}\upsilon \left(\beta \right)=1,\;\lim_{\beta \to \beta_{2}}\upsilon \left(\beta \right)=1+\frac{\sigma_{n}^{2}-\eta e_{H}}{\eta e_{H}}$, there at least exists one point which makes $\varsigma \left(\beta \right)\;=\;\upsilon \left(\beta \right)$ over the range $\beta_{1}⩽\beta ⩽\beta_{2}$. Therefore, the existence of the optimal $\beta^{*}$ has been proved. Then, we will show the uniqueness for the optimal $\beta^{*}$.

Three cases should be considered when we analyze the relationship between $\varsigma \left(\beta \right)$ and $\upsilon \left(\beta \right)$ versus β.

(1) If $\sigma_{n}^{2}<\eta e_{H}$. In this case, $\upsilon \left(\beta \right)$ is decreasing in β and $\varsigma \left(\beta \right)$ is increasing in β. Thus, $\varsigma \left(\beta \right)$ intersects $\upsilon \left(\beta \right)$ once, which is shown in Figure A1.

(2) If $\sigma_{n}^{2}=\eta e_{H}$. In this case, $\upsilon \left(\beta \right)$ is a constant value which equals 1. Thus, $\varsigma \left(\beta \right)$ intersects $\upsilon \left(\beta \right)$ once, which is shown in Figure A1.

(3) If $\sigma_{n}^{2}>\eta e_{H}$. In this case, $\upsilon \left(\beta \right)$ is also increasing in β. Let $\beta_{4}>\beta_{3}$, $u\left(\beta \right)=\ln\left(1+\frac{\left(\eta e_{H}-\eta e_{H}\beta -e_{S}\beta \right)}{\sigma_{n}^{2}\frac{\tau_{0}}{T}\left(1-\rho \left(\beta \right)\right)}-\frac{\eta e_{H}}{\sigma_{n}^{2}}\right)$, and $\varpi \left(\beta \right)=\varsigma \left(\beta \right)-\upsilon \left(\beta \right)$; then, $\varsigma \left(\beta \right)=\frac{1}{\rho \left(\beta \right)u\left(\beta \right)}$. Computing the $\varpi \left(\beta_{2}\right)-\varpi \left(\beta_{1}\right)$, we can derive that

(A14) $$\begin{array}{c} \varpi \left(\beta_{4}\right)-\varpi \left(\beta_{3}\right)=\frac{1}{\rho \left(\beta_{4}\right)u\left(\beta_{4}\right)}-\frac{1}{\rho \left(\beta_{3}\right)u\left(\beta_{3}\right)}-\left(\sigma_{n}^{2}-\eta e_{H}\right)\frac{\tau_{0}}{T}\left[\upsilon_{1}\left(\beta_{4}\right)-\upsilon_{1}\left(\beta_{3}\right)\right]\\ >\frac{1}{u\left(\beta_{4}\right)}\left[\frac{1}{\rho \left(\beta_{4}\right)}-\frac{1}{\rho \left(\beta_{3}\right)}\right]-\left(\sigma_{n}^{2}-\eta e_{H}\right)\frac{\tau_{0}}{T}\left[\frac{1-\rho \left(\beta_{4}\right)}{\eta e_{H}-\eta e_{H}\beta_{4}-e_{S}\beta_{4}}-\frac{1-\rho \left(\beta_{3}\right)}{\eta e_{H}-\eta e_{H}\beta_{3}-e_{S}\beta_{3}}\right]\\ >\frac{\sigma_{n}^{2}\frac{\tau_{0}}{T}\left(1-\rho \left(\beta_{4}\right)\right)}{\eta e_{H}-\eta e_{H}\beta_{4}-e_{S}\beta_{4}}\frac{\rho \left(\beta_{3}\right)-\rho \left(\beta_{4}\right)}{\rho \left(\beta_{3}\right)\rho \left(\beta_{4}\right)}-\frac{\frac{\tau_{0}}{T}\left(\sigma_{n}^{2}-\eta e_{H}\right)\left(1-\rho \left(\beta_{4}\right)\right)}{\eta e_{H}-\eta e_{H}\beta_{4}-e_{S}\beta_{4}}+\frac{\frac{\tau_{0}}{T}\left(\sigma_{n}^{2}-\eta e_{H}\right)\left(1-\rho \left(\beta_{3}\right)\right)}{\eta e_{H}-\eta e_{H}\beta_{3}-e_{S}\beta_{3}}\\ >\frac{\sigma_{n}^{2}\frac{\tau_{0}}{T}\left(1-\rho \left(\beta_{4}\right)\right)\left(\rho \left(\beta_{3}\right)-\rho \left(\beta_{4}\right)\right)}{\eta e_{H}-\eta e_{H}\beta_{4}-e_{S}\beta_{4}}-\frac{\frac{\tau_{0}}{T}\left(\sigma_{n}^{2}-\eta e_{H}\right)\left(1-\rho \left(\beta_{4}\right)\right)}{\eta e_{H}-\eta e_{H}\beta_{4}-e_{S}\beta_{4}}+\frac{\frac{\tau_{0}}{T}\left(\sigma_{n}^{2}-\eta e_{H}\right)\left(1-\rho \left(\beta_{3}\right)\right)}{\eta e_{H}-\eta e_{H}\beta_{3}-e_{S}\beta_{3}}\\ >\frac{\tau_{0}}{T}\left(1-\rho \left(\beta_{3}\right)\right)\frac{\sigma_{n}^{2}\left(\rho \left(\beta_{3}\right)-\rho \left(\beta_{4}\right)\right)}{\eta e_{H}-\eta e_{H}\beta_{3}-e_{S}\beta_{3}}>0. \end{array}$$

Thus, $\varpi \left(\beta \right)$ increases with β, and $\lim_{\beta \to \beta_{1}}\varpi \left(\beta \right)=-1,\lim_{\beta \to \beta_{2}}\varpi \left(\beta \right)=\;+\;\infty$, and there must exist only one β that makes $\varpi \left(\beta \right)=0$. Thus, $\varsigma \left(\beta \right)$ intersects $\upsilon \left(\beta \right)$ once, which is shown in Figure A1.

Based on the above analysis, it can be concluded that there exist unique β over the range $\beta_{1}⩽\beta ⩽\beta_{2}$. The existence and uniqueness of β has been proved. Once the $e_{H}$ and $\sigma_{n}^{2}$ are given, the relationship between $\varsigma \left(\beta \right)$ and $\upsilon \left(\beta \right)$ will be determined. From $\varsigma \left(\beta \right)=\upsilon \left(\beta \right)$, the bisection method can be adopted to acquire the optimal $\beta^{*}$. After getting the optimal $\beta^{*}$, the optimal $\alpha^{*}$ can be expressed as $\alpha^{*}=1-\beta^{*}-\frac{\tau_{0}}{T}\left(1-\rho \left(\beta^{*}\right)\right)$. According to the noise distribution characteristics of CSN and the developmental level of energy harvesting, $\sigma_{n}^{2}<\eta e_{H}$ is almost available for the communication systems generally.
